# Revealing serum lipidomic characteristics and potential lipid biomarkers in patients with POEMS syndrome

**DOI:** 10.1111/jcmm.16486

**Published:** 2021-03-28

**Authors:** Hao Zhao, Xue‐min Gao, Xin‐xin Cao, Lu Zhang, Dao‐bin Zhou, Jian Li

**Affiliations:** ^1^ Department of Hematology, Peking Union Medical College Hospital Chinese Academy of Medical Sciences and Peking Union Medical College Beijing China

**Keywords:** ferroptosis, lipidome, lysophospholipids, POEMS syndrome

## Abstract

POEMS syndrome is a rare plasma cell dyscrasia with distinct lipid metabolism abnormalities at disease onset. However, the serum lipidomic characteristics in patients with POEMS syndrome were not investigated. The study performed an untargeted lipidome screening by liquid chromatography‐tandem mass spectrometry (LS‐MS/MS) in the pre‐ and post‐treatment serum of 24 patients with POEMS syndrome, together with the serum of 24 paired healthy controls. Patients with POEMS syndrome had a distinct serum lipid composition compared with healthy controls, and a 3‐lipid model had a predictive accuracy of 93.5% in distinguishing patients and healthy controls consisting of fatty acyl 17‐oxo‐20Z‐hexacosenoic acid, phosphatidylcholine(16:0/18:1(9Z)) and sterol lipid 5b‐pregnanediol. Four lipids including 17‐oxo‐20Z‐hexacosenoic acid (r = 0.423, *P* = .040) were correlated with risk stratification, and 2 lipids including Cer(d18:0/13:0) were inversely related to serum vascular endothelial growth factor level (r=−0.465, *P* = .022). Eleven lipids were related to disease activity, including arachidonic acid which was inversely related and lysoPC(20:4) which was positively related. The study indicated a distinct lipid characteristic profile of patients with POEMS syndrome different from healthy controls and identified several lipids that may serve as potential diagnostic markers and monitors of therapeutic efficacy, as well as indicating potential metabolism pathways involved in the pathological process.

## 1 INTRODUCTION

POEMS syndrome is a rare plasma cell dyscrasia characterized by polyneuropathy, organomegaly, endocrinopathy, monoclonal gammopathy and skin changes. ^1^ The pathogenesis of POEMS syndrome remains poorly understood. Vascular endothelial growth factor (VEGF) is regarded as the cytokine that best correlates with disease activity and is responsible for clinical manifestations, such as extracellular volume overload and skin angioma.^2^, ^3^

A percentage of 37% patients with POEMS syndrome had weight loss over 10 lbs. at disease onset. ^1^ Shrinking buccal fat pads were prominent at disease onset although not regularly described in previous reports, indicating distinct lipid metabolism abnormalities. Significantly altered lipid metabolism was reported in a variety of haematological malignancies. A serum lipidomic study on patients with acute myeloid leukaemia and myelodysplastic syndromes showed that the levels of lysomonomethyl glycerophosphocholine could effectively distinguish these patients from healthy controls.^4^ A study of the serum lipidome in patients with multiple myeloma demonstrated that serum arachidonic acid levels diminished significantly in patients with monoclonal gammopathy of undetermined significance (MGUS) and multiple myeloma (MM) compared with healthy controls. Supplementation with arachidonic acid by injection in MM mice could induce ferroptosis of MM cells, indicating a new drug target for MM.^5^

However, the serum lipidomic characteristics in patients with POEMS syndrome were not investigated. Therefore, we designed this study to describe characteristics of the serum lipidome in patients with POEMS syndrome and to explore potential biomarkers in evaluating disease activity and remission.

## 2 MATERIAL AND METHODS

### 2.1 Study design

**FIGURE 1:**
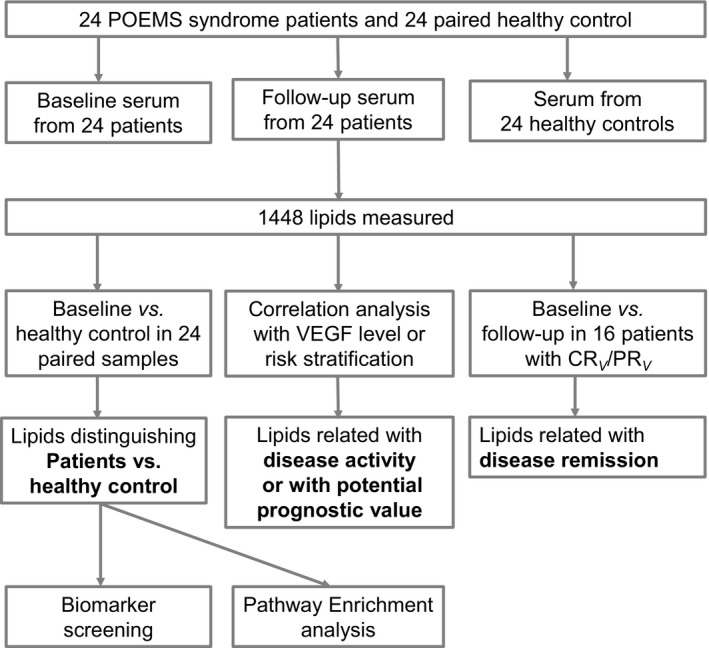
Study schema

The study schema is shown in Figure 1. A total of 24 POEMS syndrome patients were enrolled, together with 24 paired healthy controls. The baseline and follow‐up serum after at least 1 cycle of treatment was collected. By comparing serum lipid components in serum of patients vs. healthy controls, lipids altered under disease status were identified. Biomarkers contributing the most to the disease status among these lipids and enriched pathways were further identified. Lipids related to VEGF level and risk stratification were screened to identify lipids related to disease activity or possessing a potential prognostic value. Lipids related to treatment remission were identified by comparing serum lipid components in baseline vs. follow‐up serum.

#### 2.1.1 Patients

We enrolled twenty‐four patients with POEMS syndrome newly diagnosed at our hospital from September 2017 to July 2018. All patients had baseline and follow‐up serum after at least one cycle of treatment. All patients met the diagnosis criteria proposed by Dispenzieri et al^1^: (1) polyneuropathy and monoclonal gammopathy; (2) ≥1 major criteria: (a) Castleman disease; (b) osteosclerosis; and (c) elevated serum VEGF level; and (3) ≥1 minor criteria (a) organomegaly; (b) extravascular volume overload; (c) endocrinopathy; (d) skin changes; (e) papilloedema; and (f) thrombocytosis and/or polycythemia.

#### 2.1.2 Healthy controls

Twenty‐four people who underwent physical examination at our hospital in November 2018 and were age, gender, and BMI matched with 24 patients with POEMS syndrome were enrolled. BMI variances were controlled within 10%. Healthy controls were screened for the following conditions: (1) normal serum cholesterol and triacylglycerol; (2) normal fasting blood glucose; (3) without a medical history of hyperlipidemia or diabetes.

The study was approved by the Institutional Review Board of Peking Union Medical College Hospital and conducted following the ethical guidelines of the Declaration of Helsinki.

### 2.2 Clinical assessment

#### 2.2.1 Risk stratification

Based on four baseline characteristics, including age > 50 years, presence of pulmonary hypertension, presence of pleural effusion and an estimated glomerular filtration rate < 30 ml/min/1.73 m^2^, patients were stratified into three risk groups. The former three characteristics had a value of 1, and the last had a value of 2. Patients with total scores of 0, 1 and 2‐5 were assigned to the low‐, medium‐ and high‐risk groups, respectively. ^6^

#### 2.2.2 Response criteria

Complete haematological remission (CR*_H_*) was assessed by immunofixation electrophoresis and free immunoglobulin light‐chain negativity in both serum and urine. Complete VEGF remission (CR*_V_*) was defined as a decrease in serum VEGF to a normal level (< 600 pg/mL). Partial VEGF remission (PR*_V_*) was defined as a more than 50% reduction in VEGF levels in patients with baseline VEGF levels over 1 200 pg/mL. ^7^

### 2.3 Lipidomic

Serum was stored at −80℃ until analysed. Untargeted lipidomic profiling of serum samples was performed by liquid chromatography and electrospray ionization‐tandem mass spectrometry (LC‐MS/MS) as reported by Zhang et al ^8^ All MS data were acquired using the software package Xcalibur 3.0. LipidSearch software version 4.1 (Mitsui Knowledge Industry, Tokyo, Japan) was used for lipid molecular species identification in complex biological samples by referring to databases including HMDB, Lipid Maps and PubChem.

### 2.4 Statistical Analysis

LC‐MS/MS data were processed using the software package Xcalibur 3.0. The values of the untargeted lipidomic measurements were normalized to total positive or total negative ion signal. Autoscaling was performed before partial least squares discrimination analysis (PLS‐DA) and supporter vector machine (SVM) analysis.

Statistical analysis for altered lipids was performed on MetaboAnalyst 4.0. For screening significantly altered lipids between paired/unpaired serum samples, paired/unpaired two‐tailed Student's t test was used. Fold changes were computed by ANOVA. Variable importance in the projection (VIP) scores was computed with PLS‐DA. Altered lipids were screened by the following criteria: (1) adjusted *p* value by t test was significant (FDR < 0.05), (2) fold change > 2 in unpaired samples, or fold change > 2 in over 75% of paired samples. (3) VIP score > 1.

To identify lipid species contributing more to distinguishing groups, we performed automated important feature identification by SVM and performance evaluation with ROC curve analyses. The correlation between lipid species and VEGF level or risk stratification was analysed with Pearson or Spearman correlation test, respectively. Pathway enrichment analysis was performed with mummichog analysis and gene set enrichment analysis (GSEA) method.

## 3 RESULTS

### 3.1 Patient characteristics

The baseline characteristics for patients are shown in Table 1. The median age was 49 years (range, 33‐69 years). The median ONLS scores were 3 (range, 1‐10). The IgA type of the heavy chain accounted for 54.2% of the whole group. The median baseline serum VEGF level was 6 031 pg/mL (range, 1 139‐12 341 pg/mL). Low‐, medium‐ and high‐risk patients consisted of 54.2%, 33.3% and 12.5% of the group, respectively. A total of 75% patients received new‐regimen‐based therapy as a first‐line treatment, with 50% receiving bortezomib combined with dexamethasone and 25% receiving lenalidomide combined with dexamethasone. Another 20.8% of patients received autologous stem cell transplantation. Only one patient received melphalan combined with dexamethasone as a first‐line therapy.

**TABLE 1:** Clinical characteristics of 24 newly diagnosed POEMS syndrome patients

Clinical characteristics	Patients with baseline serum and healthy control(%) (N = 24)
Demographic characteristics	
Age > 50 years	33.3
Male	58.4
POEMS syndrome‐related characteristics	
Polyneuropathy (ONLS > 4)	20.8
Organomegaly	
Hepatomegaly	33.3
Splenomegaly	54.2
Lymphadenopathy	
Endocrinopathy	29.2
Diabetes	8.3
Hypothyroidism	75.0
Monoclonal gammopathy	
IgA type heavy chain	54.2
SPE > 5 g/L	12.4
BMPC > 10%	0.0
Skin lesions	
Pigmentation	91.6
Angioma	58.3
Extravascular volume overload	
Peripheral oedema	75.0
Ascites	41.7
Pleural effusion	37.5
Castleman disease	66.7 (N = 3)^a^
Papilloedema	12.5 (N = 16)^b^
Osteosclerosis	37.5
Polycythemia	16.7
Thrombocytosis	20.8
Hypoalbuminemia (Alb < 30g/L)	8.3
Renal dysfunctione GFR < 30 ml/min/ 1.73m^2^	4.2

24h urinary protein > 1g	4.2
Risk stratification	
Low risk	54.2
Medium risk	33.3
High risk	12.5
Initial treatment	
ASCT	20.8
MDex	4.2
LDex	25
BDex	50

^a^ A total of 3 out of 24 patients underwent lymph node biopsy and 2 patients were with Castleman disease.

^b^ A total of 16 out of 24 patients underwent ophthalmoscope examination and 2 patients were with papilloedema.

The lipid profile and fasting blood glucose of 24 healthy controls were in Table S1.

### 3.2 Lipidomic analysis

#### 3.2.1 Global serum lipidomic analysis

Global lipidomic analysis was performed on the pre‐ and post‐treatment serum of 24 patients with POEMS syndrome, together with the serum of their age‐, gender‐ and BMI‐paired healthy controls. A total of 1448 lipid species were quantified comprising 54 classes/subclasses from 7 main lipid categories: glycerophospholipids (32%), fatty acyls (18%), sterol lipids (14%), prenol lipids (14%), glycerolipids (10%), sphingolipids (7%) and polyketides (5%).

#### 3.2.2 Identification of characteristics of altered lipid species between patients and healthy controls

**FIGURE 2:**
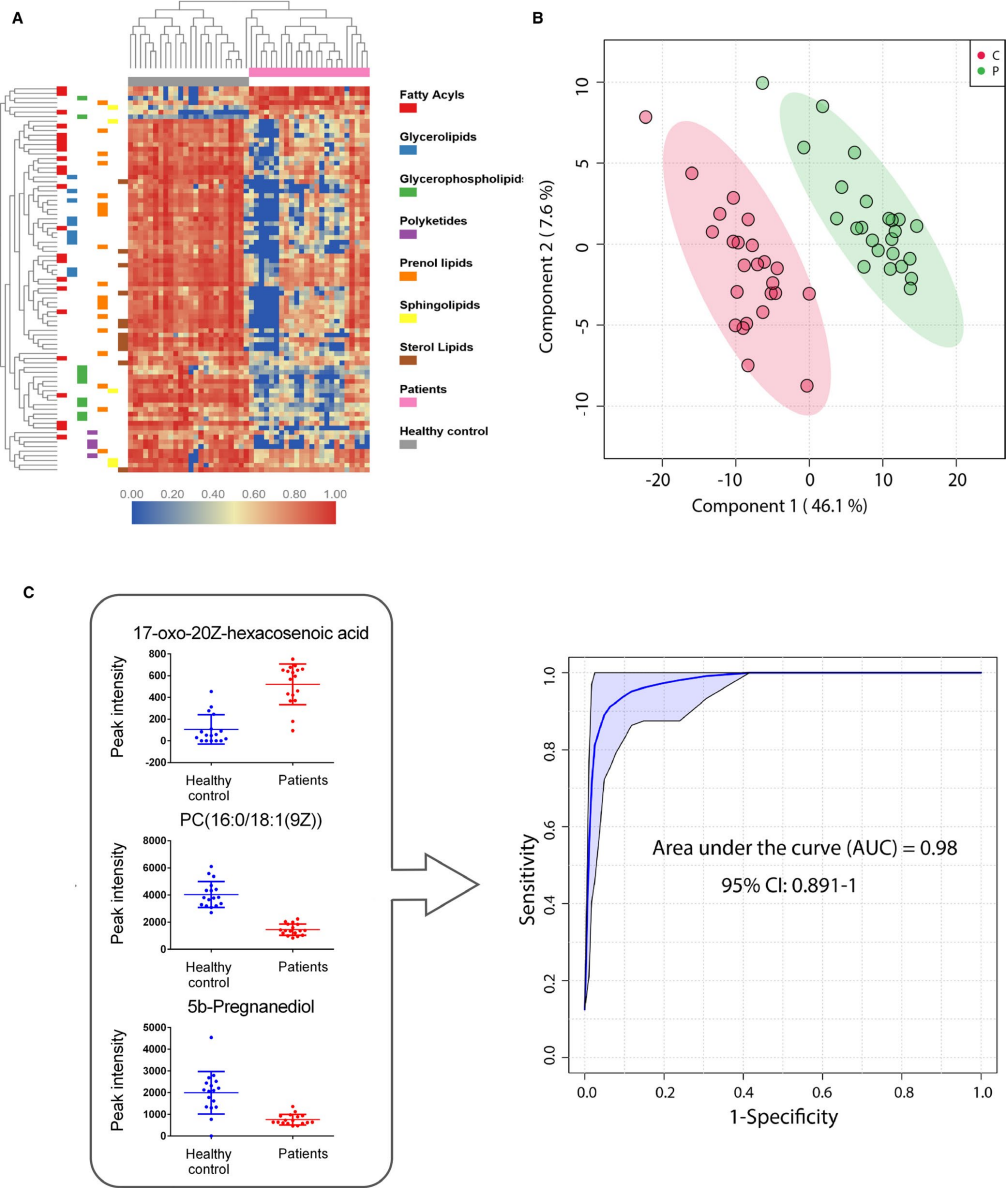
Lipidomic characteristics of POEMS syndrome patients compared with healthy controls. A, Heat map of altered serum lipids between 24 patients and paired healthy controls. The colour represents relative abundance of the lipids as compared with average level in all 48 samples, with red representing a high level and blue representing a low level. B, PLS‐DA plot. The green dots represent patients and ret dots represent healthy controls. Patients and healthy controls could be well separated by two components. C, A model identified with SVM analysis consisting of 3 lipids with a predictive accuracy of 93.5%

Patients with POEMS syndrome had a distinct serum lipid composition compared with that of healthy controls, with a significant difference in 83 metabolites (7 significantly up‐regulated and 76 significantly down‐regulated in serum samples from POEMS syndrome patients compared with healthy controls). (Figure 2A; Table S2) Patients and healthy controls could be well separated with two components in a PLS‐DA plot. (Figure 2B) Most lipids were down‐regulated in patients with POEMS syndrome than their paired healthy controls, including 86.9% (20/23) fatty acyls, 100% (10/10) glycerolipids, 80% (8/10) glycerophospholipids, 100% (4/4) polyketides, 94.7% (18/19) prenol lipids, 80% (4/5) sphingolipids and 100% (12/12) sterol lipids. Multivariate ROC curve analysis was performed to identify metabolites with the most discriminating value of patients and healthy controls. The predictive accuracy reached a level of 93.5% when only 3 lipids were enrolled consisting of fatty acyls 17‐oxo‐20Z‐hexacosenoic acid, phosphatidylcholine(16:0/18:1(9Z)) and sterol lipid 5b‐pregnanediol, with an AUC value of 0.980 (95% CI 0.891‐1.000). (Figure 2C).

Compounds with an adjusted *p* value less than 0.05 in t test between patients vs. healthy controls were enrolled in the pathway enrichment analysis. POEMS syndrome patients had serum lipids different from healthy controls enriched in the following 9 pathways: de novo fatty acid biosynthesis, vitamin E metabolism, C21‐steroid hormone biosynthesis and metabolism, bile acid biosynthesis, fatty acid activation, xenobiotics metabolism, dynorphin metabolism, vitamin D3 (cholecalciferol) metabolism and glycerophospholipid metabolism. (Table 2) Enrichment pathways by sole mummichog analysis or GSEA method were in Table S3.1, S3.2.

**TABLE 2:** The pathway enrichment analysis of altered lipids between 24 patients and paired healthy controls by mummichog algorithm and GSEA method

	Total metabolites^a^	Hits^b^	Sig_Hits^c^	Mummichog *p* value	GSEA *p* value	Combined *p* value
De novo fatty acid biosynthesis	106	14	9	0.0036	0.0308	0.0011
Vitamin E metabolism	54	18	10	0.0091	0.0156	0.0014
C21‐steroid hormone biosynthesis and metabolism	112	24	11	0.0354	0.0145	0.0044
Bile acid biosynthesis	82	48	19	0.0345	0.0167	0.0049
Fatty acid activation	74	10	6	0.0287	0.0807	0.0164
Xenobiotics metabolism	110	8	4	0.1462	0.0244	0.0237
Dynorphin metabolism	8	1	1	0.2732	0.0189	0.0323
Vitamin D3 (cholecalciferol) metabolism	16	8	4	0.1462	0.0492	0.0427
Glycerophospholipid metabolism	156	12	6	0.0769	0.1094	0.0486

^a^ Total number of metabolites in the pathway

^b^ Total number of m/z hits with *P* <.05 in t test between patients and healthy controls in the pathway

^c^ Total number of m/z hits that were considered significant based on the given cut‐off value of *P* <.00001 in mummichog analysis

#### 3.2.3 Identification of lipid species related to VEGF level and risk stratification

**FIGURE 3:**
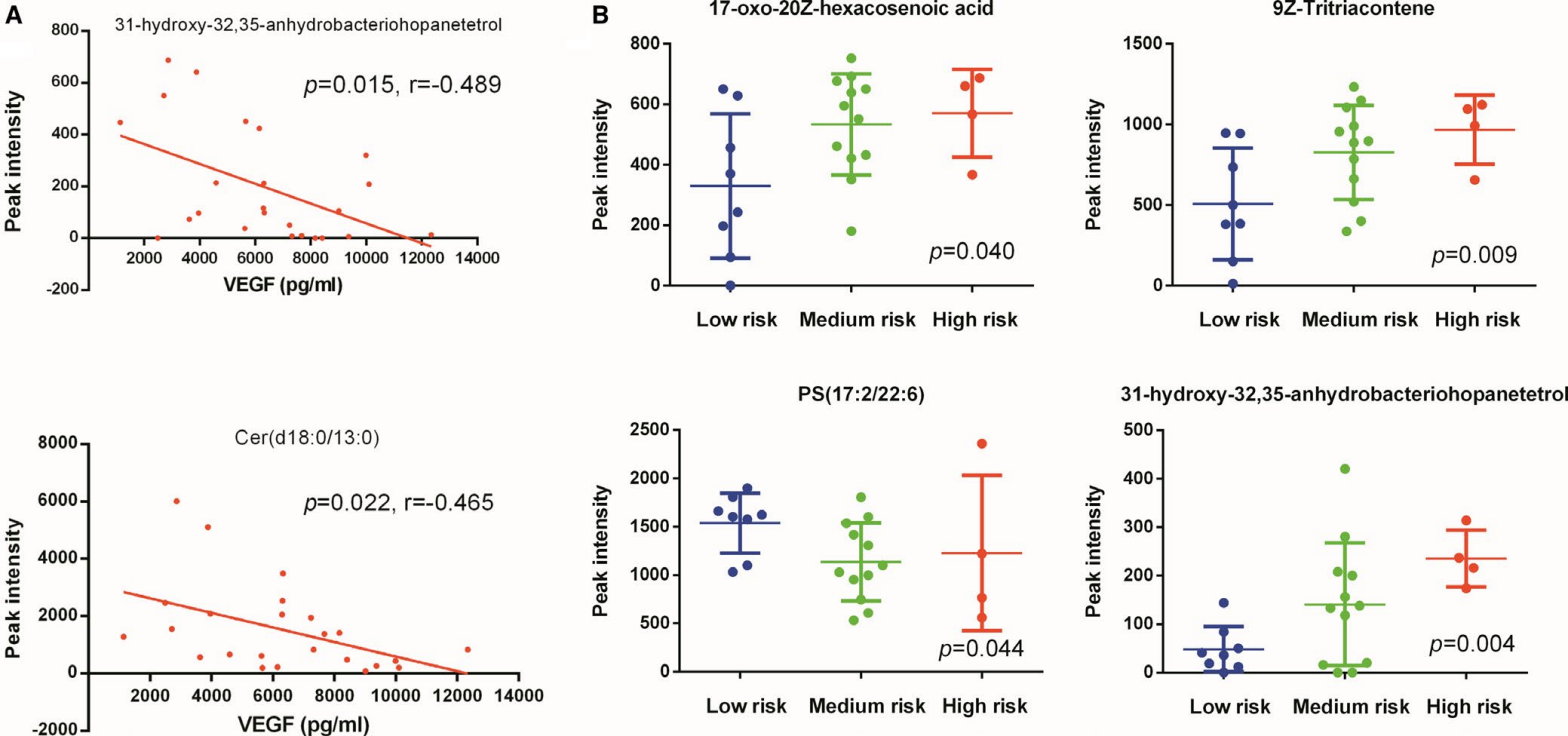
Lipids correlated with serum VEGF level or risk stratification. A, Lipids correlated with serum VEGF level. B, Lipids correlated with risk stratification

Among 83 lipids significantly altered in patients compared with healthy controls, 2 lipids were also correlated with serum VEGF levels serum of 24 patients with POEMS syndrome: prenol lipid 31‐hydroxy‐32,35‐anhydrobacteriohopanetetrol (r=−0.489, *P* =.015) and sphingolipid Cer(d18:0/13:0) (r=−0.465, *P* =.022). (Figure 3A, Table S4).

Four lipids were correlated with clinical risk stratifications including fatty acyl 17‐oxo‐20Z‐hexacosenoic acid (r = 0.423, *P* =.040), 9Z‐tritriacontene (r = 0.523, *P* =.009), PS(17:2/22:6) (r=−0.415, *P* =.044) and 31‐hydroxy‐32,35‐anhydrobacteriohopanetetrol (r = 0.570, *P* =.004). (Figure 3B; Table S5).

#### 3.2.4 Identification of lipid species related to treatment responses

After at least 1 cycle of treatment, 16 (66.7%) patients achieved CR*_V_*/PR*_V_*. Sixteen out of 24 patients were on therapy while taking follow‐up serum. Ten patients in 16 patients (62.5%) with CR*_V_*/PR*_V_* were still on bortezomib and dexamethasone therapy while taking follow‐up serum. Five out of 8 patients (62.5%) without CR*_V_*/PR*_V_* were on therapy while taking follow‐up serum, with one patient on lenalidomide and dexamethasone therapy, one on melphalan and dexamethasone therapy and another 3 patients on bortezomib and dexamethasone therapy.

**FIGURE 4:**
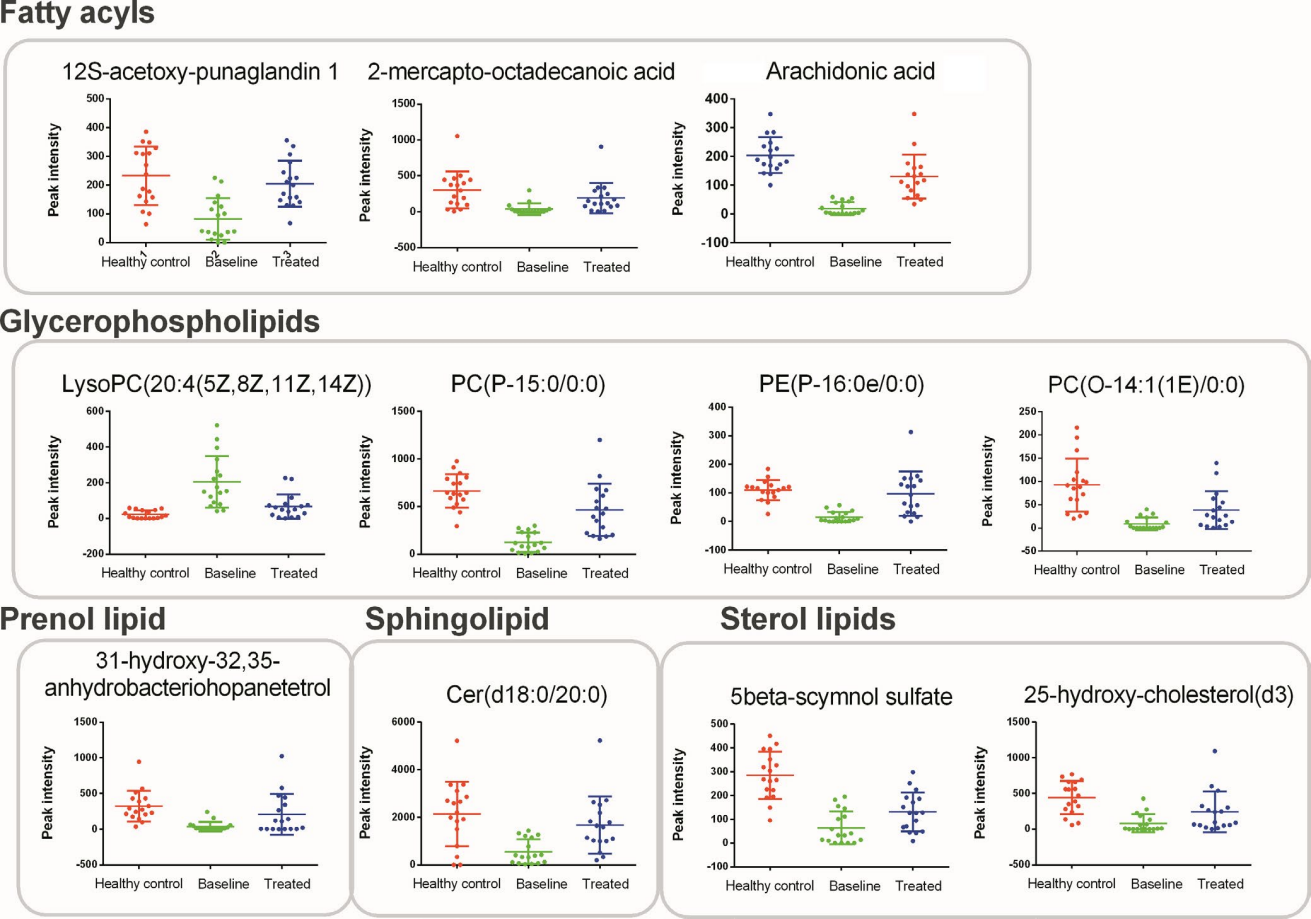
Lipids related to disease activity

To explore lipid species related to treatment responses, altered lipids between baseline vs. follow‐up samples of 16 patients with CR*_V_*/PR*_V_* were screened. Patients with responses had 23 significantly altered serum lipids, with 10 lipids diminished and 13 lipids increased after achieving CR*_V_*/PR*_V_*. (Table S6.1) Eleven lipids significantly altered between patients vs. healthy controls and were significantly altered in a backward direction after treatment were shown in Figure 4. Arachidonic acid was diminished in patients compared with healthy control (*P* <.001) and significantly increased after treatment (*P* <.001) and was regarded to be inversely related to disease activity. LysoPC(20:4), in contrary, was up‐regulated in patients (*P* <.001) and decreased after treatment (*P* =.001), therefore was positively related to disease activity. None of these 11 lipids was among 4 lipids significantly altered between baseline vs. follow‐up serum in patients without remission. (Table S6.2).

By directly comparing lipids in follow‐up serum from patients with and without CR*_V_*/PR*_V_*, we found 7 lipids that were significantly altered, comprising 2 sphingolipids, 2 glycerophospholipids, 2 sterol lipids and 1 prenol lipid. (Table S6.3) All of these altered lipids were inversely related to VEGF remission.

## 4 DISCUSSION

In the present study, we applied untargeted lipidome profiling, which yielded a broad view of the serum lipid composition of patients with POEMS syndrome.

Firstly, we explored serum lipidome of newly diagnosed POEMS syndrome patients vs. paired healthy controls. Compared with healthy controls, patients with POEMS syndrome had significantly altered serum lipidome profiles, characterized by a lower level of most altered fatty acyls and all altered glycerolipids. The pathway enrichment analysis also indicated a dysregulation in both de novo fatty acid biosynthesis pathway and fatty acid activation pathway in patients. Fatty acyls and glycerolipids, both energy storage entities, are required in large amounts during tumour cell growth and proliferation. ^9^ Patients with lung cancer were reported to have serum triacylglycerides significantly lower than in healthy controls, indicating a high energy requirement. ^10^ However, POEMS syndrome had relatively low plasma cell burden and immunoglobulin production, unparallel with the energy exhaustion status. As pathology of POEMS syndrome was not explicit, the energy exhaustion could be a joint result of increased energy consumption by plasma cells and other pathological processes.

While a total of 83 lipids were significantly altered, 3 lipids contributing the most to the difference between patients and healthy controls were identified with ROC curve analysis. As an oxo fatty acid, 17‐oxo‐20Z‐hexacosenoic acid is a marker of the inflammatory microenvironment in which unsaturated fatty acids are oxidized into oxo fatty acids. Oxo fatty acids can bind to receptors such as PPAR‐γ to further stimulate transcription of downstream redox‐regulating enzymes, modulating fatty acid storage and glucose metabolism. ^11^ As a common isoform of phosphatidylcholine, PC(16:0/18:1) was reported to serve as the endogenous ligand for the nuclear receptor PPARα in hepatocyte. ^12^ As a transcription factor regulating the expression of many genes that govern lipid metabolism, PPARα is also a drug target for hepatic steatosis. ^13^, ^14^ The depletion of serum PC(16:0/18:1) may affect lipid metabolism in POEMS syndrome patients by influencing PPARα‐regulated downstream gene expressions. In sterol lipid category, lower serum 5b‐pregnanediol level was also a main feature of POEMS syndrome patients. Pregnanediol was a metabolite of progesterone. As reported, hypogonadism is the most common endocrine abnormality in POEMS syndrome patients, in accordance with our results.^15^

Secondly, we performed a correlation analysis between serum lipids and baseline VEGF and risk stratification. Lipids inversely correlated with serum VEGF levels included Cer(d18:0/13:0), which was also lower in baseline serum of patients vs. healthy controls. Ceramide was reported to have a proapoptotic effect in the pathological malignant condition. Decreased serum ceramide may contribute to more vigorous growth of monoclonal plasma cells and production of VEGF in POEMS syndrome patients which needs further validation. Besides, 17‐oxo‐20Z‐hexacosenoic acid was positively correlated with baseline clinical risk stratification, along with another 3 lipids. This indicates a potential prognostic value of serum lipidomic features, as a molecular prognosis biomarker based on baseline serum is still absent for POEMS syndrome patients.

Thirdly, we compared baseline and treated samples split by with CR*_V_*/PR*_V_* or not. Arachidonic acid diminished in patients and rebounded after disease remission. A previous study found that arachidonic acid was significantly reduced in the serum of patients with MGUS and MM and that supplementation with arachidonic acid in MM mice by injection could induce ferroptosis of MM cells, revealing a novel apoptotic pathway for drug targets. ^5^ Whether a decrease in patients’ serum arachidonic acid level affects survival of monoclonal plasma cells by interfering with cell ferroptosis is worth further investigation. LysoPC(20:4) significantly increased in patients and decreased after remission, paralleled with disease activity. As lysophosphatidylcholine was reported to mediate demyelination and pericyte loss in brain, it may play a role in peripheral neuropathy resulting from demyelination of peripheral nerves in POEMS syndrome. ^16^, ^17^ Besides, the value of the other 9 lipids related to disease activity on disease activity monitoring should be validated in larger cohorts.

We conducted an untargeted lipidome screening of the serum of patients with POEMS syndrome. Our study indicated a distinct lipid characteristic profile of patients with POEMS syndrome different from healthy controls and identified several lipids that may serve as potential diagnostic markers and monitors of therapeutic efficacy, as well as indicating potential metabolism pathways involved in the pathological process.

## CONFLICT OF INTEREST

The authors declare no competing financial interests.

## AUTHOR CONTRIBUTION

**Hao Zhao:** Conceptualization (equal); Data curation (equal); Formal analysis (equal); Investigation (equal); Methodology (equal); Project administration (equal); Software (equal); Writing‐original draft (equal); Writing‐review & editing (equal). **Xue‐min Gao:** Conceptualization (equal); Data curation (equal); Investigation (equal); Methodology (equal); Project administration (equal); Writing‐original draft (equal); Writing‐review & editing (equal). **Xin‐xin Cao:** Conceptualization (equal); Methodology (equal); Project administration (equal); Resources (equal); Supervision (equal). **Lu Zhang:** Conceptualization (equal); Methodology (equal); Resources (equal); Supervision (equal). **Dao‐bin Zhou:** Conceptualization (equal); Resources (equal); Supervision (equal). **Jian Li:** Conceptualization (lead); Data curation (lead); Formal analysis (lead); Funding acquisition (lead); Project administration (lead); Resources (lead); Supervision (lead); Validation (lead); Writing‐review & editing (lead).

## Supporting information

Table S1‐S6

## Data Availability

The data that support the findings of this study are available from the corresponding author upon reasonable request.
